# Supplementary material for: Study on Bulk-Surface Transport Separation and Dielectric Polarization of Topological Insulator Bi1.2Sb0.8Te0.4Se2.6
Source: Molecules. 2024 Feb 15;29(4):859. doi: 10.3390/molecules29040859 (PMC10893539; doi:10.3390/molecules29040859)
Supplement: Supplementary file 1 [file molecules-29-00859-s001.zip › molecules-2844580-supplementary.pdf]

Supplemental Material

Study on Bulk-Surface Transport Separation and Dielectric Polarization of Topological Insulator $Bi_{1.2}Sb_{0.8}Te_{0.4}Se_{2.6}$

Yueqian Zheng[a], Tao Xu[a], Xuan Wang[a, *], Zhi Sun[a], Bai Han[a]

[a] Department of Electric engineering, Harbin University of Science and Technology, 52 Xuefu Rd, Nangang, Harbin, Heilongjiang, 150080, China

*To whom correspondence may be addressed: Xuan Wang(wangxuan@hrbust.edu.cn)

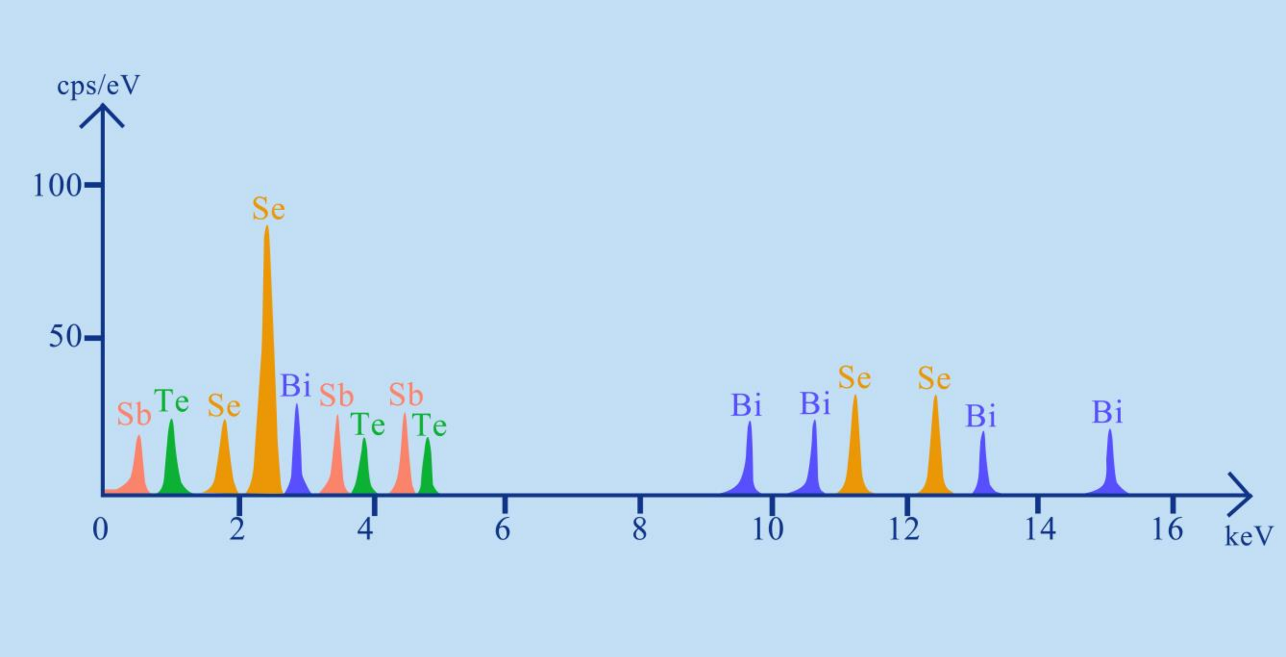

Figure S1. Energy-dispersive spectrum of the BSTS sample.

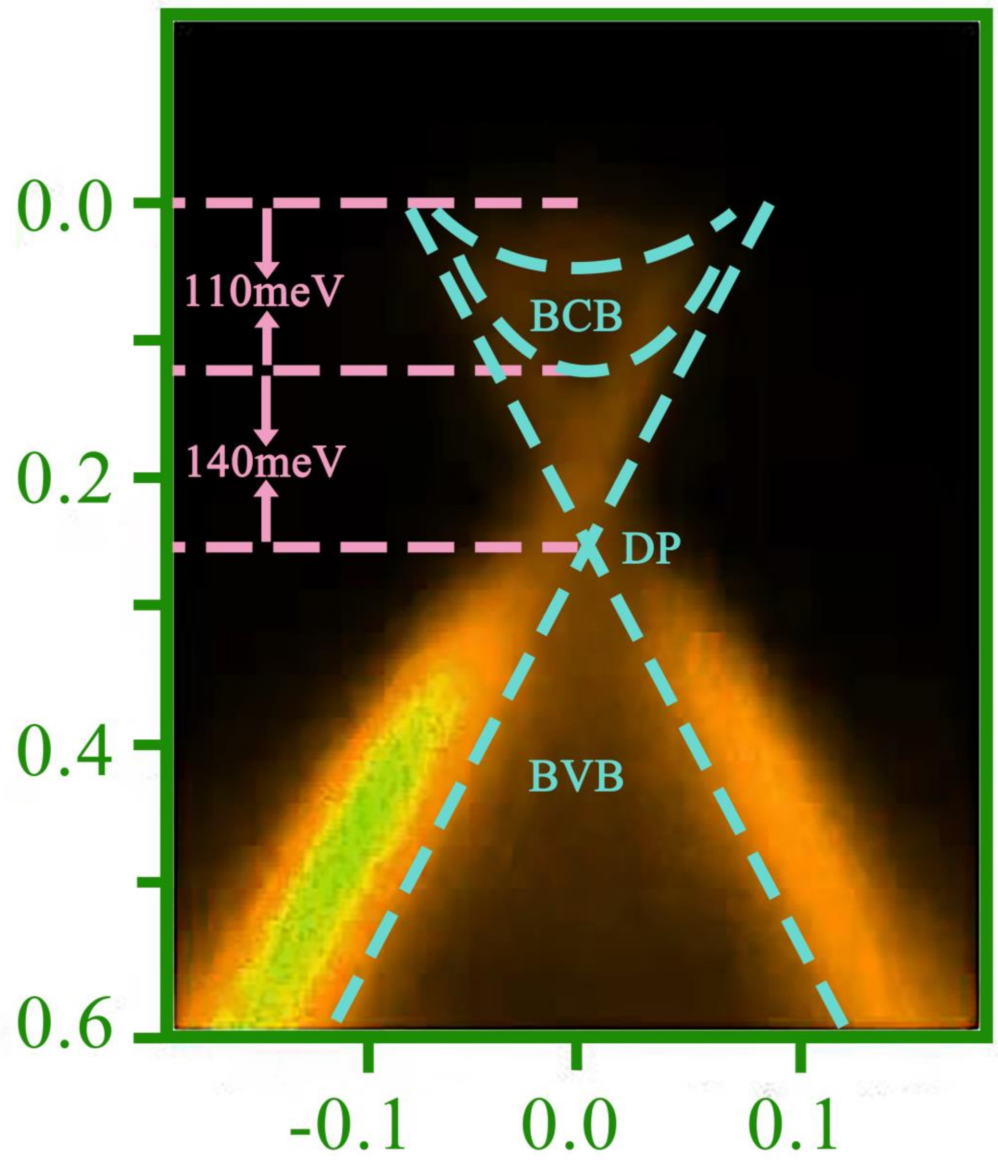

Figure S2. Angular-resolved photoemission spectrum of the BSTS sample, exhibiting the characteristic band structure of the "Dirac cone".

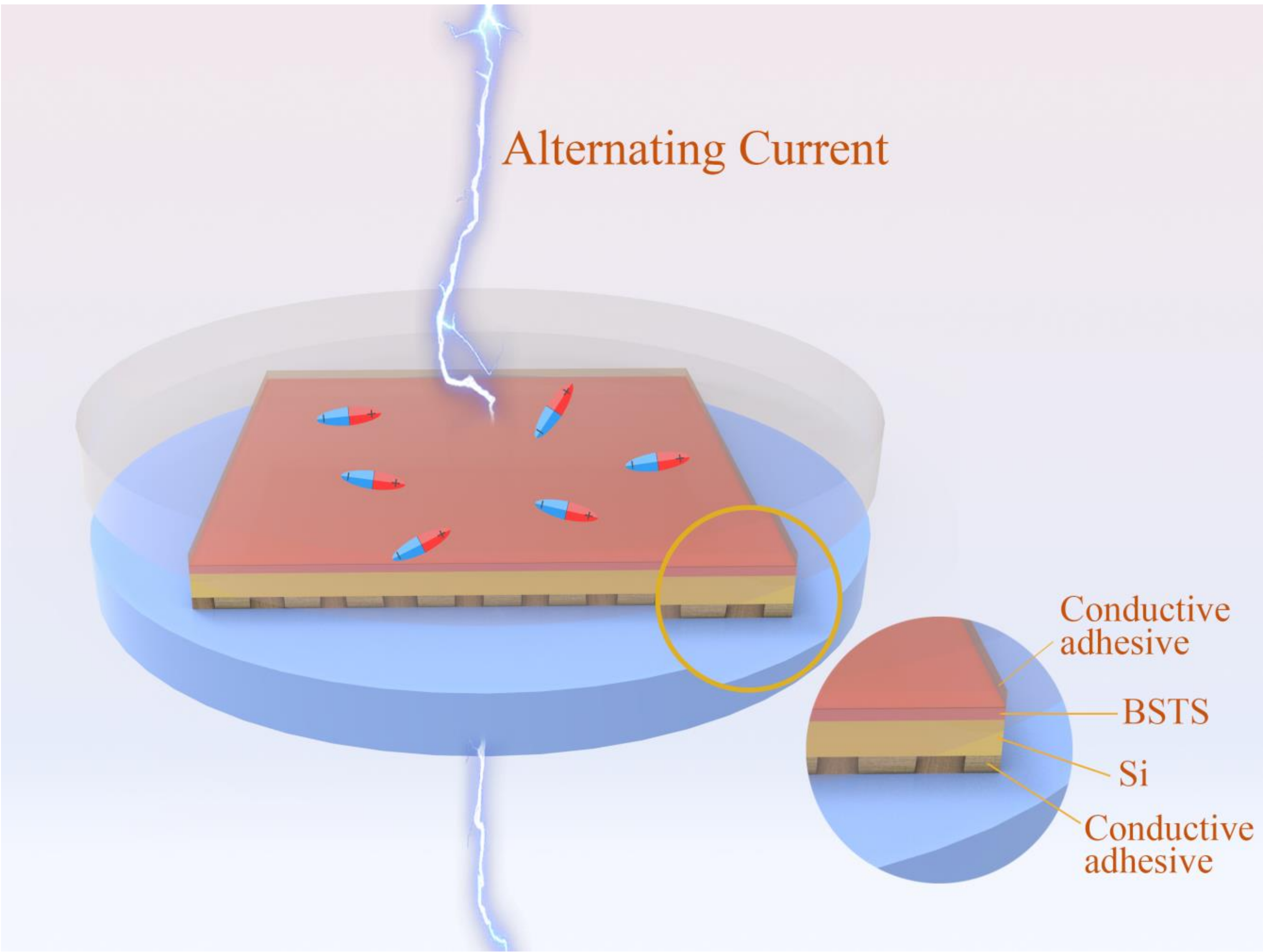

Figure S3. Testing principle of Broadband Dielectric Spectroscopy Instrument.
